# *NTRK1* rearrangement in colorectal cancer patients: evidence for actionable target using patient-derived tumor cell line

**DOI:** 10.18632/oncotarget.5494

**Published:** 2015-10-12

**Authors:** Su Jin Lee, Gang Gary Li, Seung Tae Kim, Min Eui Hong, Jiryeon Jang, Nara Yoon, Soo Min Ahn, Danielle Murphy, Jason Christiansen, Ge Wei, Zachary Hornby, Dong Woo Lee, Joon Oh Park, Young Suk Park, Ho Yeong Lim, Sung No Hong, Seok-Hyeong Kim, Won Ki Kang, Keunchil Park, Woong Yang Park, Kyoung-Mee Kim, Jeeyun Lee

**Affiliations:** ^1^ Division of Hematology-Oncology, Department of Medicine, Samsung Medical Center, Sungkyunkwan University School of Medicine, Seoul, Korea; ^2^ Ignyta Inc, San Diego, California, USA; ^3^ Department of Pathology and Translational Genomics, Samsung Medical Center, Sungkyunkwan University School of Medicine, Seoul, Korea; ^4^ Samsung Electrics, Seoul, Korea; ^5^ Division of Gastroenterology, Department of Medicine, Samsung Medical Center, Sungkyunkwan University School of Medicine, Seoul, Korea; ^6^ Samsung Genome Institute, Seoul, Korea; ^7^ The Innovative Cancer Medicine Institute, Samsung Medical Center, Sungkyunkwan University School of Medicine, Seoul, Korea

**Keywords:** colorectal cancer, *NTRK1* rearrangement, TRKA immunohistochemistry

## Abstract

**Background:**

We have investigated the incidence of *NTRK1* rearrangements in metastatic gastrointestinal cancer patients and demonstrated the potential for clinical response of these patients to targeted therapy.

**Methods:**

We prospectively collected tumor tissue specimens for one year and simultaneously generated patient-derived tumor cells (PDCs). Specimens were initially screened for TrkA protein expression using TrkA immunohistochemistry (IHC). In the case of TrkA IHC positive results, samples were further examined by fluorescence *in situ* hybridization (FISH) and next generation sequencing (NGS) to confirm the presence of *NTRK1* rearrangements.

**Results:**

From January 2014 to December 2014, a total of 74 metastatic colorectal cancer (CRC) patients and 66 gastric cancer (GC) patients were initially screened by TrkA IHC. Two of the 74 CRC patients (2.7%) and one of the 66 GC patients (1.5%) were positive for TrkA expression by IHC. All three IHC positive cases had evidence of *NTRK1* rearrangements by FISH. NGS was performed on the 3 IHC positive cases and confirmed TPM3-*NTRK1* rearrangements in the two CRC cases. One GC patient with TrkA expression by IHC did not harbor an *NTRK1* rearrangement. PDCs established from the *NTRK1* positive CRC patients were positive for the *NTRK1* rearrangement. Entrectinib, a pan-TRK inhibitor, profoundly inhibited cell proliferation of *NTRK1*-rearranged PDCs with such inhibition associated with inactivation of TrkA, and down-regulation of downstream signaling pathways.

**Conclusion:**

TrkA IHC is an effective, initial screening method for *NTRK1* rearrangement detection in the clinic. Inhibition of the TrkA kinase is a promising targeted therapy for cancer patients whose tumors harbor a *NTRK1* rearrangement.

## INTRODUCTION

The Neurotrophic Tyrosine Kinase Receptor 1 gene (*NTRK1*) encodes the TrkA receptor, which is a member of the TRK (tropomyosin-receptor kinase) family of receptor tyrosine kinases (RTKs) that also includes TrkB (encoded by *NTRK2*) and TrkC (encoded by *NTRK3*) [1]. In normal physiological conditions, TrkA is activated through NGF-mediated dimerization, which induces autophosphorylation of specific tyrosine residues and transphosphorylation of a series of substrates, leading to activation of PI3K/AKT, Ras/MAPK and PLC-γ pathways [2]. The first *NTRK1* gene fusion was identified in a colon cancer specimen, which had sequences from the *TPM3* (non-muscle tropomyosin) gene [3, 4]. Subsequently, *NTRK1* fusions have been detected at a frequency of 12% in papillary thyroid cancer with *TPM3*-*NTRK1* being the most common gene rearrangement [5–7]. More recently, *NTRK1* rearrangements have been further identified in other tumor types, including Spitzoid melanoma (16.4%, 23/140) [8], intrahepatic cholangiocarcinoma (3.6%, 1/28) [9], glioblastoma (1.1%, 2/185) [10], pediatric high grade glioma (7.1%, 8/112) [11] and sarcoma (1%, 1/103) [12]. This established evidence in various cancer types suggests that oncogenic rearrangement of *NTRK1* might occur at low frequency across many other cancer types [13].

We previously reported the presence of a very rare *ROS1* rearrangement in gastric cancer (GC) with an incidence of 0.8% using immunohistochemistry (IHC) screening [14]. Since then, we have incorporated IHC to identify very rare rearrangements in various tumor types. Given the rapidity, broad applicability and low costs of IHC in clinical diagnostic labs, we investigated the prevalence of *NTRK1* rearrangements in gastrointestinal and colorectal cancers with TrkA IHC. Break-apart fluorescence *in situ* hybridization (FISH) was performed to confirm *NTRK1* rearrangement in the IHC-positive cases. Additionally, *NTRK1* rearrangements were further analyzed with NGS to identify the specific fusion partner.

We identified 2 CRC patients that have *TPM3-NTRK1* gene rearrangements out of 74 CRC patients in this study (2.7% prevalence). Patient derived cells (PDC) from one of these patients demonstrate a targeted tumor cell response, in both 2-D and 3-D experiments, to the TrkA inhibitor, entrectinib. These data demonstrate that *NTRK1* gene rearrangements in CRC patients are sensitive to treatment with entrectinib.

## RESULTS

### Patients’ characteristics

A total of 66 patients with GC and 74 patients with colorectal cancer (CRC) were included in this study. Of 66 patients with GC, 45 patients (68.2%) were male with median age of 56 years (range, 30–80 years). Most of the patients were stage III/IV (93.8%) and poorly differentiated (80.3%). Most common metastatic sites were as follows in the order of frequency: peritoneal seeding (47.0%), liver (27.3%), lymph node (22.7%), ovary (9.1%), lung (4.5%) and bone (4.5%). Detailed characteristics of patients are shown in Table 1. In the CRC group, median age was 60 years (range, 19–82 years) and the male-to-female ratio was 0.85. Primary site of disease is colon in 47 patients (63.5%) and rectum in 25 patients (33.8%). Initial stage was mostly stage III (33.8%) or IV (60.8%), and primary resection was performed in 59 patients (79.7%). Most patients showed good to moderate differentiation (83.8%). *KRAS* mutation was detected in 36.6%, and *BRAF* mutation was detected in 1.7%. Most common metastatic sites were as follows: liver (47.3%), lung (37.8%), lymph node (29.7%), peritoneal seeding (20.3%), ovary (12.2%) and bone (5.4%).

**Table 1:** Characteristics of GI cancer patients (*N* = 140)

	Gastric cancer (*N* = 66)		Colon Cancer (*N* = 74)
Characteristic	Number (%)	Characteristic	Number (%)
**Age (years)** Median, (range)	56 (30–80)	**Age (years)** Median, (range)	60 (19–82)
**Sex** Male Female	45 (68.2) 21 (31.8)	**Sex** Male Female	34 (45.9) 40 (54.1)
**Primary site** Upper 1/3 Middle 1/3 Lower 1/3 Whole	15 (23.8) 17 (27.0) 29 (46.0) 2 (3.2)	**Primary site** Appendix A-Colon T-Colon D-Colon S-Colon Rectum	2 (2.7) 14 (18.9) 6 (8.1) 5 (6.8) 22 (29.7) 25 (33.8)
**Primary resection** Yes No	23 (34.8) 43 (65.2)	**Primary resection** Yes No	59 (79.7) 15 (20.3)
**Initial stage** III IV Unknown	13 (20.0) 48 (73.8) 4 (6.1)	**Initial stage** III IV Unknown	25 (33.8) 45 (60.8) 4 (5.6)
**Differentiation** Well to moderately differentiated Poorly differentiated	13 (19.7) 53 (80.3)	**Differentiation** Well to moderately differentiated Poorly differentiated Mucinous	62 (83.8) 5 (6.8) 4 ((4.1)
		**KRAS mutation (*N* = 71)**	26 (36.6)
		**BRAF mutation (*N* = 58)**	1 (1.7)
**Immunohistochemistry** Trk (*N* = 66) HER2 (*N* = 64) cMET (*N* = 62)	2 (3.0) 6 (9.4) 8 (12.9)	**Immunohistochemistry** Trk (*N* = 74) HER2 (*N* = 70) cMET (*N* = 68)	1 (1.7) 3 (4.1) 027 (39.7)
**Site of metastasis** Peritoneal seeding Lymph node Liver Ovary Lung Bone	31 (47.0) 15 (22.7) 18 (27.3) 6 (9.1) 3 (4.5) 3 (4.5)	**Site of metastasis** Liver Lung Lymph node Peritoneal seeding Ovary Bone	35 (47.3) 28 (37.8) 22 (29.7) 15 (20.3) 9 (12.2) 4 (5.4)

### Identification of TrkA-positive cases

**Figure 1:**
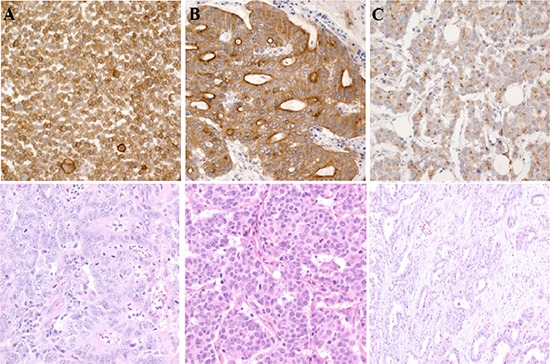
Trk protein expression by IHC in A. patient #1 B. patient #2 and C. patient #3

**Figure 2:**
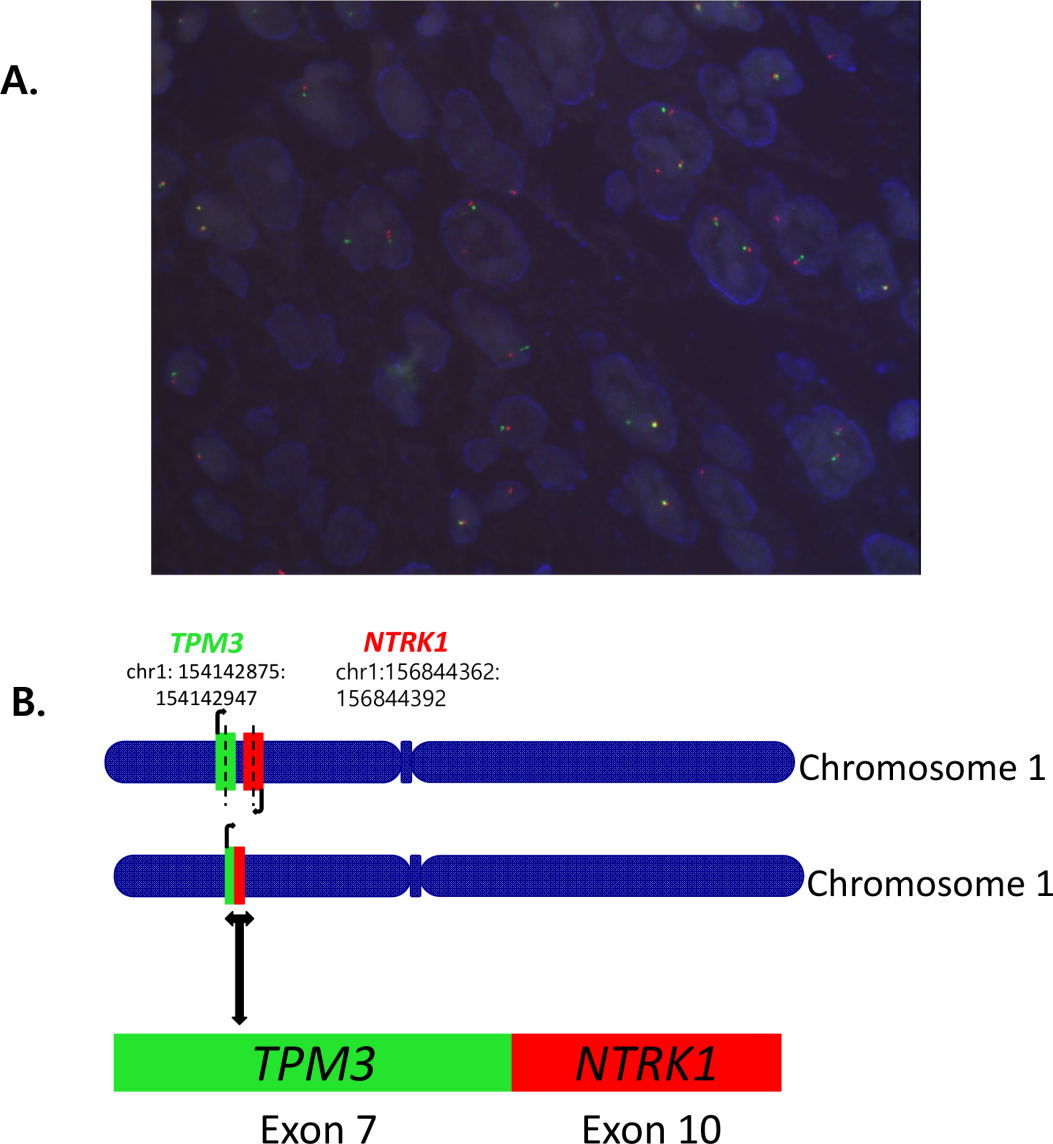
A. Fluorescence *in situ* hybridization (FISH) for break-apart of *NTRK1* gene and B. *TPM3-NTRK1* fusion with the 5′ end of *NTRK1*, including the kinase domain, starting at exon 10 fused to exon 7 of TPM3 by NGS

With five primary antibodies, we set up IHC with different conditions and found panTRK (C17F1) from Cell signaling as one of the most sensitive and specific primary antibody and adopted this for screening. We found two out of 74 CRCs (2.7%) and one out of 66 GCs (1.5%) positive for Trk IHC. Representative photograph of Trk protein expression by IHC is shown in Figure 1. In these Trk(+) cases, FISH analysis showed break-apart of *NTRK1* gene in three out of four examined cases; 68% (Patient #1, Figure 2), 20% (Patient #2), and 20% (Patient #3) of examined tumor cells.

### Characteristics of *NTRK1*-rearranged cancer patients

**Patient #1**. The first case is a 72-year-old male patient who presented initially with stage III *KRAS* wild-type, *BRAF* wild-type ascending colon cancer in year 2013. He underwent right hemicolectomy, and the pathology revealed AJCC stage IIIB. He recurred with multiple cervical, left supraclavicular, retroperitoneal, intra-abdominal lymph node metastases after 5 cycles of adjuvant XELOX (capecitabine and oxaliplatin) chemotherapy. The chemotherapy regimen has been switched to irinotecan/capecitabine chemotherapy, which stabilized the disease for 4 months, but then the disease progressed to the lymph nodes. At this time, core biopsy from a supraclavicular lymph node was performed and, after tumor confirmation, we generated PDCs from the patient. The patient has progressed to cetuximab/irinotecan chemotherapy and is now deceased. The tumor has been confirmed by NGS to harbor the previously observed *TPM3-NTRK1*. fusion with the 5′ end of *NTRK1*, including the kinase domain, starting at exon 10 fused to exon 7 of *TPM3* (see Figure 2).

**Patient #2**. The second patient is a 71-year-old female who was diagnosed in 2014 with multiple axillary lymph nodes and multiple liver metastases from rectal cancer. The tumor was KRAS and BRAF wild-type. After six cycles of first-line Avastin/FOLFIRI chemotherapy (fluorouracil/irinotecan/bevacizumab), tumor progressed to multiple lower abdominal lymphadenopathies. The patient has completed 12 cycles of second-line FOLFOX (oxaliplatin, 5-FU, leucovorin) chemotherapy with stable disease. This patient was confirmed to have *TPM3-NTRK1.* fusion by NGS (identical to patient #1, see Figure 2).

**Patient #3**. The third patient is a 68-year-old male who presented with metastatic gastric cancer with metastases to retroperitoneal lymph nodes and supraclavicular lymph nodes. The patient has HER2 negative adenocarcinoma. The patient has received 12 cycles of FOLFOX and 8 cycles of irinotecan at the time of this writing. Although this patient had Trk(+) by IHC and FISH, NGS failed to find *NTRK* rearrangements because of poor quality of RNA.

### Confirmation of *TPM3*-*NTRK1* fusion as an oncogenic driver *in vitro*

**Figure 3:**
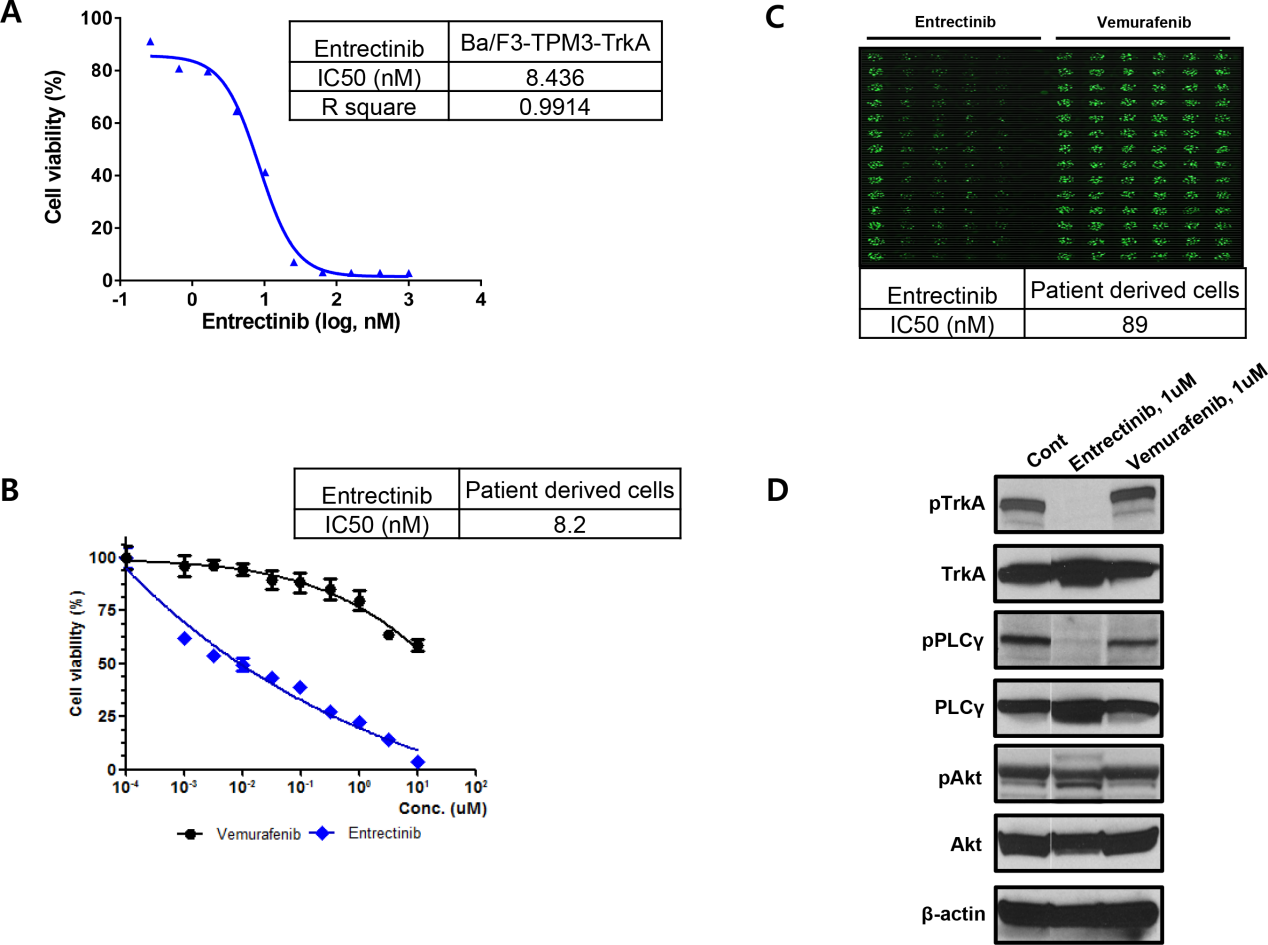
The conventional MTT assay for entrectinib using **A.** Ba/F3 cell and **B.** patient derived cells, **C.** The evaluation of IC50 for entrectinib using the high-throughput 3-dimensional culture system and **D.** The western blot for TrkA phosphorylation and targeted downstream pathways.

*TPM3*-*NTRK1* cDNA was cloned from KM12 cells by RT-PCR and *TPM3*-TrkA protein was expressed in Ba/F3 cells using lentiviral expression system. Two weeks after the Ba/F3 cells were cultured in mIL3 and puromycin, the cells were further cultured in RPMI media without mIL3 and puromycin for additional 6 weeks. The cells showed robust growth without mIL3, confirming its oncogenic driving ability. Expression of *TPM3*-TrkA protein was confirmed by Flow-cytometry and qPCR. Subsequently, Ba/F3-*TPM3*-TrkA cells were treated with entrectinib for 3 days and the cell viability was accessed using CellTiter-Glo^®^ reagents. As expected, entrectinib inhibits the cell proliferation and the IC50 value is the same as what was obtained from PDCs (Figure 3A and 3B). Similar western blot profiles were observed in these cells (data not shown)

### *TPM3*-*NTRK1* rearrangement positive patient derived cells from metastatic lymph nodes of CRC patient #1

Patient-derived tumor cells (PDCs) were established from metastatic lymph nodes from patient #1. After cytological confirmation, the PDCs were treated with entrectinib. Entrectinib is a potent, selective, orally available, ATP-competitive inhibitor of tyrosine kinases TrkA, TrkB, TrkC, *ROS1* and *ALK* (encoded by *NTRK1*, *NTRK2*, *NTRK3*, *ROS1* and *ALK* genes, respectively). The presence of *NTRK1* rearrangement was confirmed in PDCs by genomic profiling (not shown). This tumor is negative for *ROS1* and *ALK* gene rearrangement or protein expression (data not shown). The IC50 from the conventional MTT assay demonstrated 0.0082 uM entrectinib (Figure 3B). Using high-throughput 3-dimensional culture system, the IC50 was 0.089 uM for entrectinib in *NTRK1* rearranged colon cancer PDCs (Figure 3C). Western blot revealed that entrectinib potently inhibited TrkA phosphorylation and targeted downstream pathways (Figure 3D).

## DISCUSSION

In this study, the incidence of TrkA (encoded by *NTRK1*) IHC positivity is 1.5% in gastric cancer and 2.7% in colorectal cancer patients by IHC. Both of the TrkA IHC positive CRC cases were confirmed to have *TPM3*-*NTRK1* rearrangements. However, the two TrkA IHC+ GC patients were not positive for *NTRK1* FISH. From this data, we have demonstrated that IHC is a viable screening strategy for *NTRK1* rearrangements in CRC. Tumor cells derived from one of the two TrkA IHC+ CRC patients were sensitive to a potent pan-TrkA inhibitor, entrectinib, with inhibition of downstream pathways, suggesting that *NTRK1* rearrangements in non-lung or non-thyroid cancer can be a potent actionable genetic aberration as in NSCLC [18].

Since the first discovery of the *TPM3*-*NTRK1* fusion in a colon cancer sample in 1982 [3], clinical development targeting this molecular subset has not been highlighted in metastatic colon cancer. Recently, Ardini et al used TrkA IHC to identify one *TPM3*-*NTRK1* rearrangement out of 66 patients tested [18], demonstrating a prevalence of 1.5% that is comparable to what is observed in the cohort described here (2.7%). Both of TrkA CRC cases reported here were *KRAS* wild-type and *BRAF* wild-type, whereas the case reported by Ardini et al was *KRAS* wild-type. [18] However, in one GC, we failed to prove *NTRK* rearrangements by NGS due to poor quality of RNA and the existence of *NTRK1* rearrangement in GC remained to be proven in the near future.

The clinical benefit from targeting *NTRK1* fusions is being tested in ongoing phase I/II entrectinib clinical trials. Entrectinib is an orally available, potent and selective ATP-competitive pan-Trk, *ROS1* and *ALK* inhibitor with IC50 activities against TrkA. TrkB and TrkC of <10 nM [19]. In this report, we successfully established patient tumor cells from a *TPM3*-*NTRK1* fusion CRC patient and demonstrated that these cells were potently inhibited by entrectinib at an IC50 of 8.2nM in the conventional MTT assay and 89 nM in 3-dimentional high throughput drug screening system. Although more data needs to be accumulated to demonstrate the concordance between responses to therapy in patient derived tumor cells and the actual tumor response in the corresponding patient, it is very promising that the tumor cells from patients with *TPM3*-*NTRK1* fusion gene dramatically responded to entrectinib. Of note, we also demonstrated that the four TrkA IHC+ patients were all negative for rearrangements in *NTRK2*, *NTRK3*, *ROS1* and *ALK*, precluding alternative target effects besides TrkA (*NTRK1* gene). Re-expression of *TPM3*-*NTRK1* in Ba/F3 further confirms that TPM3-TrkA fusion protein along is capable to be oncogenic driver. Therefore Inhibition of the TrkA kinase is a promising targeted therapy for cancer patients whose tumors harbor a *NTRK1* rearrangement.

## MATERIALS AND METHODS

### Patients

This study included GC and CRC patients who were on palliative chemotherapy at Samsung Medical Center, Seoul, Korea, between January 2014 and December 2014. All study participants provided written informed consent before study entry. Briefly, patients with metastatic solid tumors who may be eligible for clinical trial enrollment were eligible to enter the study. Patients also consented for *in vitro* establishment of patient-derived cells (PDCs) for research use if tissue was available.

### Immunohistochemistry for Trk

Briefly, 4 μm tissue sections were deparaffinized and rehydrated, and antigens were retrieved for 40 min in a citrate buffer (pH 6.1) at 95°C. We used panTRK (C17F1 Rabbit mAb; 1: 100 dilution; Cell Signaling) as the primary antibody incubating 15 min with Bond-max autoimmunostainer (Leica Biosystem, Melbourne, Australia). DAB was used as the chromogen, and the sections were counterstained with hematoxylin. For positive control, we used KM12 cell line, which is known to have a *TPM3*-*NTRK1* rearrangement.

### Fluorescence *in situ* hybridization (FISH)

For analysis of *NTRK1* gene rearrangements by fluorescence *in situ* hybridization (FISH), in cases of positive results of TrkA overexpression by IHC, we used ZytoLight SPEC *NTRK1* Dual Color Break Apart Probe for the detection of translocations involving the *NTRK1* gene at 1q23.1 (ZytoVision, Bremerhaven, Germany) according to the operating instructions. With the use of appropriate filter sets, the interphases of normal cells or cells without a translocation involving the 1q23.1 band, two green/orange fusion signals appear. A 1q23.1 locus affected by a translocation is indicated by one separate green signal and one separate orange signal. A threshold of 15% nuclei positive for break apart signals was used to establish the cut off for positive FISH.

### Next generation sequencing (NGS)

FFPE tissue sections of 10μM thickness were microdissected to enrich tumor sample, as identified on a proximal H&E slide, prior to nucleic acid extraction. Next generation sequencing (NGS) of extracted RNA was performed to identify gene rearrangements in *NTRK1*, *NTRK2*, *NTRK3*, *ROS1* and *ALK* [15]. Briefly, the test consists of library preparation using anchored multiplex PCR coupled to sequencing on an Illumina MiSeqDx system.

### Ba/F3-*TPM3*-TrkA cell generation and proliferation assay

To generate Ba/F3 cells expressing *TPM3*-TrkA, *TPM3*-TrkA cDNA was cloned from KM12 cells by RT-PCR and inserted into a lentiviral vector pVL-EF1a-MCS-IRES-Puro (BioSettia, San Diego, CA). After confirmation by direct sequencing, VSVG-pseudo-typed lentivirus containing *TPM3*-TrkA cDNA was introduced into the murine IL-3 dependent pro-B cell Ba/F3. The transduced Ba/F3 cells were selected at 1 ug/mL of puromycin in the murine IL-3 containing RPMI and 10% FBS media for two weeks. The stable cell pools were further selected in RPMI and 10% FBS media without murine IL-3 for 4 weeks. To evaluate the anti-proliferative activity of entrectinib, Ba/F3-*TPM3*-TrkA cells were seeded at 5,000 cells per well in 96-well assay white plates (Costar #3610) with different concentrations of Entrectinib (0 to 1 μM) at duplicates. Three days after incubation, cell viabilities were measured by luciferase-based ATP level detection using CellTiter-Glo^®^ reagents (Promega, Madison, WI, USA) and IC50s were determined by 4-parameter curve fit with variable slope.

### Patient-derived cell culture and reagents

Patient derived cell cultures were established as previously described [16]. Tumors were removed using core biopsy from a supraclavicular lymph node and then homogenized. Extracted cells were cultured in RPMI media supplemented with 10% fetal bovine serum, 0.5 g/ml of hydrocortisone (Sigma Aldrich), 5 g/ml of insulin (PeproTech, Rocky Hill, NJ, USA), 5 ng of EGF and FGF (PeproTech). Vemurafenib was purchased from Selleck Chemical (Houston, TX, USA). Entrectinib was provided by Ignyta, Inc. (San Diego, CA, USA). After pathologic confirmation, cells were seeded at 1 × 10^6^ cells/10 mm dishes or 5000 cells/well/96well plate and treated with 1 μM of entrectinib. Treated cells were incubated for 72 hours at 37°C in a humidified atmosphere of 5% carbon dioxide. These conditions were used for analysis of Immunoblotting and cell proliferation inhibition assays, which were run in triplicate. Entrectinib is a TrkA, TrkB, TrkC, *ALK*, *ROS1* inhibitor that is currently undergoing a phase 1/2 clinical trial in the US (http://www.clinicaltrials.gov; NCT02097810) and a first in human study in Italy [14]. Inhibition of tumor derived cell line proliferation was determined using CellTiter-Glo^®^ reagents (Promega, Madison, WI, USA) according to the manufacturer's protocol. For 3-dimensional drug screening system, we utilized the S+ Chip Analyzer (Samsung Electro-Mechanics Co., Ltd, South Korea) to analyze the image of the cells grown in 3D droplets on micropillars as previously described [17]. Briefly, for intensity measurement, the software defines the boundary of analysis as each micropillar edge and calculates mean intensity by dividing total green fluorescence intensities (represented by 8 bit values) in the micropillar with the total pixels of the micropillar (boundary of analysis). In case of the area, the software counts the number of pixel whose intensity is more than background (established as 30, in the 8 bit green range) and calculates area of the colonies [17].

### Immunoblot analysis

Total proteins from patient tumor derived cell lines were isolated using RIPA buffer (Sigma-Aldrich, St. Louis, MO, USA) containing a protease inhibitor cocktail (Roche, Mannheim, Germany) and phosphatase inhibitor cocktail (Roche). Protein concentrations were determined according to Bradford procedure using a Quick Start Bradford Protein Assay (Bio-Rad, Hercules, CA, USA). Thirty μg of proteins were subjected to 10% SDS-polyacrylamide gel electrophoresis, and electro-transferred onto nitrocellulose membranes. The membranes were blocked with 5% nonfat dry milk in Tris-buffered saline containing 0.1% v/v Tween 20, and probed overnight at 4°C with specific antibodies: pTrakA(Tyr490), TrkA, pPLCγ1(Tyr783), PLCγ1, pAkt(Ser473) and Akt from Cell Signaling Technology (Beverly, MA, USA) and beta actin from Sigma Aldrich. Horseradish peroxidase-conjugated anti-rabbit or mouse IgG (Vector, Burlingame, CA, USA) were used as secondary antibodies. Immune complexes were visualized by enhanced chemiluminescence using ECL Western Blotting Substrate (Thermo Scientific, Rockford, IL, USA).
